# The Medical Pocket Book, for 1835

**Published:** 1835-01-01

**Authors:** 


*The Medical Pocket Book, for* 1835. By JOHN FOOTE, Jun. London: small 8vo. pp. 166.

*The Medical Almanack, for* 1835. London. 12mo. pp. 72.

THE Medical Pocket Book is a handsome little work, and contains many useful memoranda. About a hundred pages consist of a diary and case-book, leaving, as we think, too little room for the various lists required. Mr. FOOTE has given a good abstract (intermingled with some errors) of the nature of our medical corporations and societies; and his account of new medicines will be serviceable. We wish he had entered into this part of pharmacology more fully, and had omitted the abstract of the pharmacopœia.

The Medical Almanack is very rich in lists, but they are unfortunately often obsolete. Thus we find, at p. 23, Sir Wm. Franklin (long since deceased,) put down as belonging to two societies; and, at p. 28, Dr. Bradley and Mr. Chevalier are enumerated among the officers of the Westminster General Dispensary. The list, too, of the College of Physicians, given at p. 16 et seq. is not the last one. There is a strange mistake at p. 72; Dr. Jenner is stated to have died in 1798—now this is the year in which he promulgated his great discovery: his death took place in 1823.

Both these works will be improved by being enlarged. A copious abstract of the laws relating to medical practitioners would be useful; (the Almanack gives half a page;) and the tax tables are much required. When the young doctor finds his income rising to a point which to the esoteric is £100 a year, but to the exoteric £1000, a cab is not merely a comfort, but, according to many grave and learned authorities, a necessary of life, more indispensable than coals or candles; and how pleasant, then, to know beforehand how much he is required to contribute to the state from the superfluous wealth indicated by his vehicle and its tiger!

The plan, both of the Pocket Book and the Almanack, is good; but Mr. Foote's work is certainly superior in the execution.

